# Polarization-Insensitive Broadband THz Absorber Based on Circular Graphene Patches

**DOI:** 10.3390/nano11102709

**Published:** 2021-10-14

**Authors:** Jiajia Qian, Jun Zhou, Zheng Zhu, Zhenzhen Ge, Shuting Wu, Xiaoming Liu, Jian Yi

**Affiliations:** 1 Terahertz Research Center, School of Electronic Science and Engineering, University of Electronic Science and Technology of China, Chengdu 610054, China; 202022021718@stu.uestc.edu.cn (J.Q.); 18256001987@163.com (Z.Z.); edward_gzz@163.com (Z.G.); 13870175230@163.com (S.W.); 2 Key Laboratory of Terahertz Technology, Ministry of Education, Chengdu 610054, China; 3 School of Physics and Electronic Information, Anhui Normal University, Wuhu 241002, China; xiaoming.liu@ahnu.edu.cn; 4 Anhui Provincial Engineering Laboratory on Information Fusion and Control of Intelligent Robot, Wuhu 241002, China; 5 Key Laboratory of Marine Materials and Related Technologies, Ningbo Institute of Materials Technology and Engineering, Chinese Academy of Sciences, Ningbo 315201, China; yijian@nimte.ac.cn; 6 Zhejiang Key Laboratory of Marine Materials and Protective Technologies, Ningbo 315201, China

**Keywords:** terahertz, absorber, graphene, metasurface, polarization-insensitive device

## Abstract

A polarization-insensitive broadband terahertz absorber based on single-layer graphene metasurface has been designed and simulated, in which the graphene metasurface is composed of isolated circular patches. After simulation and optimization, the absorption bandwidth of this absorber with more than 90% absorptance is up to 2 THz. The simulation results demonstrate that the broadband absorption can be achieved by combining the localized surface plasmon (LSP) resonances on the graphene patches and the resonances caused by the coupling between them. The absorption bandwidth can be changed by changing the chemical potential of graphene and the structural parameters. Due to the symmetrical configuration, the proposed absorber is completely insensitive to polarization and have the characteristics of wide angle oblique incidence that they can achieve broadband absorption with 70% absorptance in the range of incident angle from 0° to 50° for both TE and TM polarized waves. The flexible and simple design, polarization insensitive, wide-angle incident, broadband and high absorption properties make it possible for our proposed absorber to have promising applications in terahertz detection, imaging and cloaking objects.

## 1. Introduction

Terahertz (THz) [1] wave usually refers to the electromagnetic wave in the frequency range of 0.1–10 THz (wavelength of 3 mm to 30 μm). The THz band is located between the microwave and infrared regions in the electromagnetic spectrum, it has been historically known as the “THz gap” due to the lack of efficient emitters and receivers operating in this regime at ambient temperature. However, a series of recent breakthroughs have helped to bridge this gap [2,3], to the point that nowadays it is a field of intense and multidisciplinary research with many applications coming into reality in numerous sectors, like chemistry, biomedicine, astronomy, national defense, security inspection, remote sensing and wireless communications [4,5,6,7,8,9]. The focus on this spectrally rich region is attributed to the highly coherent and non-ionizing nature of THz radiation, wide unallocated frequency bands, distinctive wavelengths and their penetration through a significant depth of dielectric materials. To fully exploit the potential of THz wave, THz devices that can efficiently manipulate the phase, amplitude and polarization of THz waves are required for the above-mentioned myriad of applications. Therefore, THz devices such as splitters, filters, absorbers, modulators, encoders, switches, polarizers and lens have been studied intensively in the THz regime [10,11,12,13]. In recent decades, a considerable effort has been made to develop THz devices using metasurfaces, which have attracted much attention because of their special physical properties and important applications in electromagnetic wave manipulation. Along with the urgent need for practical applications, this research field will be further developed in depth in the future.

The study of THz absorbers is critical to realize many potential THz applications, including imaging, detection and stealth technology [14,15,16]. The mechanism of the absorber is to reduce the reflection and transmission of the incident wave as much as possible, and the electromagnetic energy is dissipated mainly in the form of electric loss and magnetic loss. Researchers have done a number of work on the absorbers, especially the metamaterial-based THz absorbers. The first perfect absorber based on metamaterial was proposed by Landy et al. [17] in 2008, which consists of a sandwich structure of metal resonant ring, dielectric substrate and metal wire and can achieve perfect absorption in specific frequencies based on LC resonance of the resonant ring. After that, Hou-Tong Chen [18] proposed a perfect THz absorber with sandwich structure, but the bottom layer replaced the metal wire with a whole metal plate, which could nearly perfectly reflect electromagnetic waves, so now the similar three-layer absorbers employ the metal plate directly. In addition, metal square rings, circular split rings and composite metamaterials structures are presented to attain double narrow band, multi narrow band and broadband absorption, and rotational symmetry structures are proposed to achieve polarization independent characteristic [19,20,21,22,23,24,25]. However, the traditional absorbers are made of metal materials that have no tunable flexibility, which limits the practical applications of the devices. Considering different systems and device flexibility, it is very necessary to develop tunable THz devices. In recent decades, a considerable effort has been made to develop tunable THz devices using different materials. Combining metasurfaces with doped semiconductors to achieve electric control of THz wave transmissions is one of the earliest methods to construct THz devices [26]. Then, photoinduced THz devices based on metasurface-bulk semiconductor hybrid structure with phase modulation was developed [27]. Later, the tunable characteristics of phase transition materials such as ${\mathrm{VO}}_{2}$ films and organic ferroelectrics were applied to thermally and optically control THz devices [28,29]. Furthermore, graphene and other two-dimensional materials were applied in tunable THz devices [30,31].

Graphene, a two-dimensional honeycomb material [32], composed of a single layer of tightly packing carbon atoms, which can support surface plasmons in the mid-infrared and THz bands [33,34]. Combining with its excellent mechanical and electromagnetic tunable properties, graphene has become one of the most promising materials for tunable THz absorbers. The conductivity and carrier mobility of graphene can be dynamically adjusted by chemical doping or electrostatic doping [35,36], so as to realize the dynamic tunability of the absorption frequency. Based on this fact, the THz absorption in graphene sheet has been widely studied both theoretically and experimentally [37,38]. However, from the results in the literature, it is very difficult to achieve more than 90% absorptance in a broadband with unpatterned graphene. At present, a variety of types of THz absorbers based on graphene periodic patterns have been demonstrated, such as graphene disks, ribbons, cross shaped, circular splitting rings, square loops, fishnets, anti-dots and other structures have realized the perfect absorption of THz waves [39,40,41,42,43,44,45,46,47]. Furthermore, in order to achieve the effect of broadband absorption, absorbers composed of metasurface with hybrid patterned graphene have been proposed. For example, Y. Li et al. [48] proposed an absorber with graphene rings and disks in periodically staggered arrangement, which provides a good reference for broadband absorber design. L. Ye et al. [49] proposed an absorber with electric-connected hybrid square/disk/loop patterned graphene metasurface. These absorbers are all based on single-layered graphene arrays, which can be easily fabricated by the chemical vapor deposition (CVD) and lithography techniques [50,51]. However, considering the influence of fabrication accuracy caused by complex patterns, it is recommended to use simple structures in graphene absorber design. Besides, multilayer graphene structures have been proposed to acquire better capacity, Rishi Mishra et al. [52] designed a hybrid, tunable multilayered THz absorber integrating with cascaded graphene frequency selective surfaces (FSSs) and dielectric layers, which has extremely high absorptance over a wide frequency range, but the manufacturing process of these multilayer structures is difficult.

Based on the existing research results, in this paper we proposed a broadband THz absorber with simpler structure and more convenient fabrication. It uses the sandwich structure mentioned above and only relies on the metasurface of single-layer circular graphene patches to obtain broadband absorption, and the maximum bandwidth of 90% absorptance can reach 2 THz. Furthermore, the proposed absorber is insensitive to polarization and has weak dependence on incident angle. It still has high bandwidth absorption for wide range of incident angle under TE and TM polarized wave incidence conditions. First, the structural design and theoretical analysis of the proposed absorber are introduced in this paper. Next, COMSOL Multiphysics software is used for modeling and numerical simulation. The principle of broadband absorption is analyzed by electric field distributions, and the polarization insensitivity of the absorber is verified. Meanwhile, the broadband absorption of the absorber is explained by impedance matching according to the effective medium theory. Then, the influence of structural parameters and chemical potential on the absorption properties are further studied, so as to obtain the optimized structural parameters. Finally, the effects of different incident angles on the absorption sensitivity under TE and TM polarized waves incidence are studied and discussed.

## 2. Design of Structure

**Figure 1:**
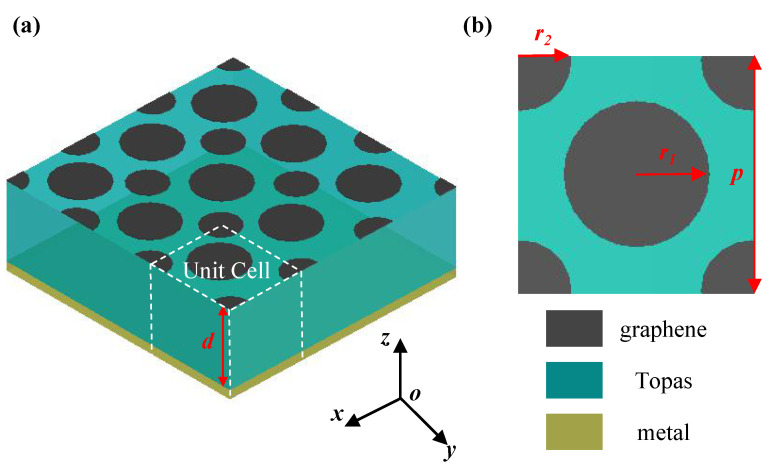
(**a**) The three-dimensional structure schematic diagram of the proposed absorber, it is based on isolated circular graphene patches with different radii in periodically staggered arrangement. (**b**) The top view of one unit cell. *d* is the substrate thickness, $r_{1}$ and $r_{2}$ are the radii of the patches with two sizes, and *p* represents the period of the unit cell.

The proposed broadband THz absorber is based on isolated circular graphene patches with different radii in periodically staggered arrangement, and its three-dimensional structure is illustrated in Figure 1a. The absorber is a three-layer structure consisting of circular graphene patches at the top, the middle dielectric substrate and a grounded metal plate at the bottom. Figure 1b shows the top view of one unit cell, showing that the graphene patches on the top layer has two radii, $r_{1}$ and $r_{2}$, respectively, and the period of the unit cell is *p*. The dielectric substrate is polyethylene cyclic olefifin copolymer Topas with a relative permittivity of 2.35 and thickness of *d*.

As a two-dimensional planar material, graphene can be characterized by surface conductivity. The surface conductivity σ of graphene is composed of two parts: the conductivity $\sigma_{\mathrm{intra}}$ contributed by the electron photon scattering in the band and the conductivity $\sigma_{\mathrm{inter}}$ contributed by the interband transition of carriers, and it can be expressed by Kubo formula when the magnetic field is neglected [53,54],

(1) $$\sigma_{\mathrm{intra}}\left(\omega ,\mu_{c},\Gamma ,T\right)=\frac{je^{2}}{\pi \hbar^{2}\left(\omega -\;j2\Gamma \right)}\int_{0}^{\infty }\xi \left[\frac{\partial f_{d}\left(\xi ,\mu_{c},T\right)}{\partial \xi }-\frac{\partial f_{d}\left(-\xi ,\mu_{c},T\right)}{\partial \xi }\right]d\xi ,$$

(2) $$\sigma_{\mathrm{inter}}\left(\omega ,\mu_{c},\Gamma ,T\right)=-\frac{je^{2}\left(\omega -\;j2\Gamma \right)}{\pi \hbar^{2}}\times \int_{0}^{\infty }\frac{f_{d}\left(-\xi ,\mu_{c},T\right)-f_{d}\left(\xi ,\mu_{c},T\right)}{{\left(\omega -\;j2\Gamma \right)}^{2}-4\xi /\hbar^{2}}d\xi ,$$

(3) $$\sigma \left(\omega ,\mu_{c},\Gamma ,T\right)=\sigma_{\mathrm{intra}}\left(\omega ,\mu_{c},\Gamma ,T\right)+\sigma_{\mathrm{inter}}\left(\omega ,\mu_{c},\Gamma ,T\right),$$

where $f_{d}\left(\xi ,\mu_{c},T\right)={\left(e^{\left(\xi -\mu_{c}\right)/k_{B}T}+1\right)}^{-1}$ is the Fermi–Dirac distribution, ω is the angular frequency, Γ is the scattering rate, and $\Gamma ={\left(2\tau \right)}^{-1}$ (τ is the electron relaxation time), ξ is the electron kinetic energy, $\mu_{c}$ is the chemical potential, $k_{B}$ is the Boltzmann constant, *T* is the absolute temperature, *e* is the electron charge and *ℏ* is the reduced Planck constant. The analytical solution of intraband conductivity of graphene can be obtained by calculating the integral on the right side of Equation (1),

(4) $$\sigma_{\mathrm{intra}}\left(\omega ,\mu_{c},\Gamma ,T\right)=\frac{je^{2}k_{B}T}{\pi \hbar^{2}\left(\omega +\;j2\Gamma \right)}\times \left[\frac{\mu_{c}}{k_{B}T}+2\ln\left(e^{-\mu_{c}/k_{B}T}+1\right)\right].$$

In the low THz frequency band and at room temperature, the interband conductivity is far less than the intraband conductivity, so the graphene conductivity σ can be approximately expressed as

(5) $$\sigma \approx \sigma_{\mathrm{intra}}=\frac{je^{2}k_{B}T}{\pi \hbar^{2}\left(\omega +\;j2\Gamma \right)}\times \left[\frac{\mu_{c}}{k_{B}T}+2\ln\left(e^{-\mu_{c}/k_{B}T}+1\right)\right].$$

It can be seen from the above formula that the conductivity of graphene depends on the frequency, scattering rate, temperature and chemical potential. The chemical potential depends on the carrier density and can be controlled by gate voltage, electric field, magnetic field and/or chemical doping [55].

In this study, COMSOL Multiphysics software was used to carry out the numerical simulations to verify the performance of the device. The frequency domain solver is used in the simulation, the boundary of *x* and *y* directions is set as periodic boundary and the port perpendicular to *z* direction is set as Floquet port. Because the thickness of graphene is only 0.35 nm, the amount of mesh computing is heavy and the calculation speed is very slow, graphene is modeled as a surface current boundary with the thickness of 0 to improve the simulation efficiency. Then, the surface current density of graphene can be expressed by its surface conductivity: J=σ∗E. We set the ambient temperature at 300 K, the value of τ and $\mu_{c}$ as 0.1 ps [56,57] and 0.75 eV, respectively, and dimensional parameters of design are as follows: p=40μm, d=21μm, $r_{1}=13\;\mu m$, $r_{2}=10\;\mu m$. According to the previous analysis, it is known that the bottom metal plate acts as a perfect reflective layer in the structure. In simulation, the metal plate is equivalent to the ideal conductor boundary, but in actual processing, its thickness is greater than the skin depth of the THz wave in order to fully reflect the incident wave. The absorptance of an absorber can be expressed as A=1−T−R, where $T=|S_{21}{|}^{2}$ represents the transmittance and $R=|S_{11}{|}^{2}$ represents the reflectance. Due to the bottom metal can close to perfectly reflect the incident wave, that is, T≈0, therefore, the absorptance of the device can be expressed as A=1−R. Obviously, the performance of the device is only determined by the reflection coefficient, so it is necessary to reduce the reflection to improve the absorptance. We assume that the incident plane electromagnetic wave propagates along the *z* direction and the electric field vector is in the *x* direction.

## 3. Results and Discussion

**Figure 2:**
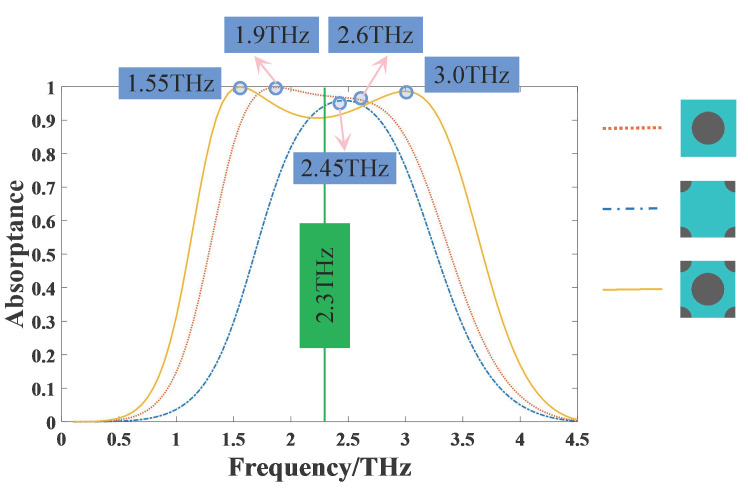
The absorptance of different graphene patterns, such as larger disk array (red dot), smaller disk array (blue dash-dot), and hybrid disk array (orange solid).

To study the broadband absorption properties of THz, three different graphene patterns were simulated, including a graphene circular patch located only at the center of square unit cell (larger disk array), quarter of graphene circular patches located at the four vertices of square unit cell (smaller disk array) and the structure proposed in this paper combined the above two (hybrid disk array). Their absorptances are compared as shown in Figure 2. The simulation results show that the smaller disk array presents a single narrower absorption band with a peak at ~2.45 THz, while the larger disk array presents a broader absorption band with relatively higher absorptance. They provide 90% absorption bandwidth of 0.62 THz and 1.4 THz, respectively, with the corresponding frequency ranges from 2.15 THz to 2.77 THz and from 1.43 THz to 2.87 THz. By combining these two structures, the 90% absorption bandwidth can be significantly increased to 2 THz, ranging from 1.34 THz to 3.36 THz. There are two absorption peaks at 1.55 THz and 3.0 THz, both have absorptance above 99%. For the hybrid disk array, although the absorptance at the center frequency (about 2.3 THz) decreases slightly, the absorption bandwidth is greatly increased and the overall absorption is still considerable, so this sacrifice is worth the effort.

**Figure 3:**
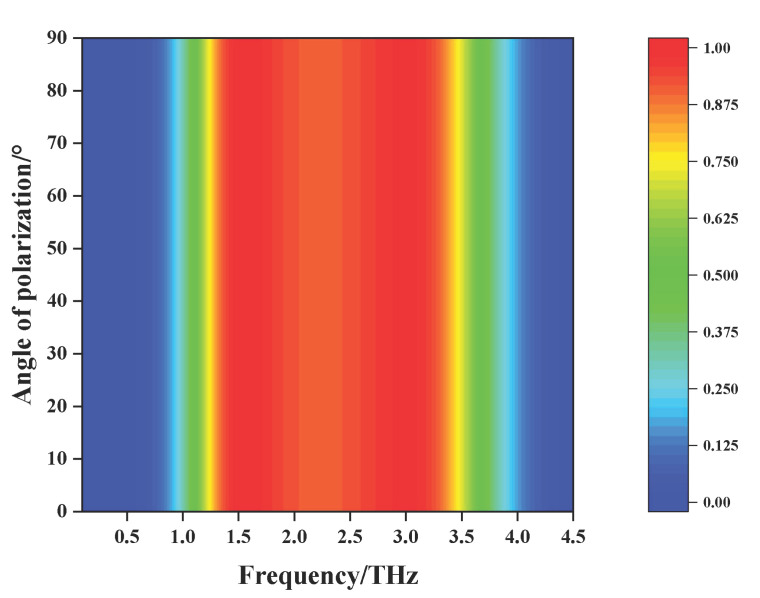
The dependence of the absorptance on the polarization angle in the case of normal incidence.

All the above simulations were performed under TE polarized waves with normal incidence. The polarization insensitivity is very important for an absorber in many cases. The structure of the designed absorber is rotationally symmetric, so it has equivalent resonance structure for incident TE and TM polarized THz waves, that is, it is independent of polarization. Figure 3 displays the relationship between the absorptance and the polarization angle in the case of normal incidence. Obviously, when the polarization angle increases from 0° to 90°, the absorptance of the absorber is completely coincident, indicating that the absorber has perfect polarization insensitive characteristics, which well verifies that the simulation results are in good agreement with the design principles.

**Figure 4:**
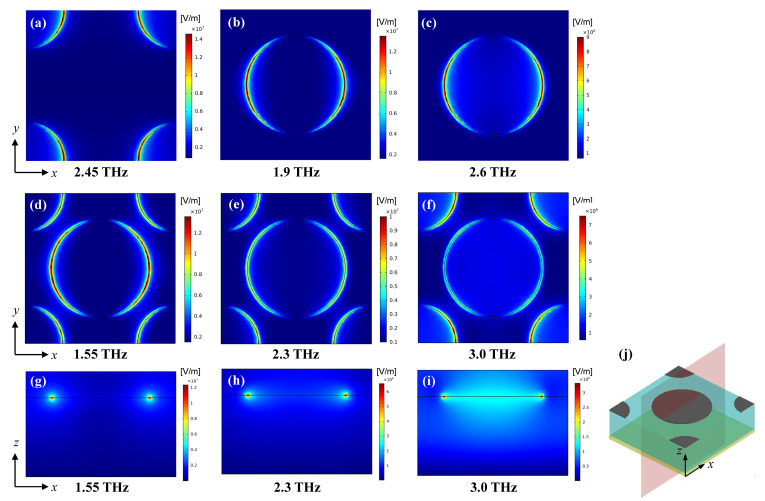
The electric field distributions under TE polarized waves. (**a**) The electric field distribution at 2.45 THz for the smaller disk array in *x-y* plane; (**b**,**c**) The electric field distributions at 1.9 THz and 2.6 THz for the larger disk array in *x-y* plane; (**d**–**f**,**g**–**i**) are the electric field distributions at 1.55 THz, 2.3 THz and 3.0 THz for the hybrid disk array in *x-y* plane and *x-z* plane, respectively; (**j**) indicates the cross section in *x-z* plane.

In order to understand the working mechanism of the absorber, we observed the electric field distributions at some selected frequencies which have been specially marked in Figure 2. As shown in Figure 4a, for the smaller disk array, when it works at the resonant frequency 2.45 THz, the electric field is mainly distributed on the surface of graphene disks, which means that the incident waves excite localized surface plasma (LSP) resonances and result in an absorption peak. As shown in Figure 4b,c, for the larger disk array, when it works at a higher frequency 2.6 THz, the electric field distribution is similar to the above mentioned LSP mode; when it works at a lower frequency 1.9 THz, the electric field is mainly distributed at the edges of graphene disks, which means that there are extra resonances caused by the coupling between the graphene disks. Because when the center distance is fixed, the larger the radius, the smaller the spacing, and the easier the coupling occurs between them, so we think the coupling is the main reason why the larger disk array presents a broader absorption band. The LSP and coupling modes correspond to the higher and lower frequencies, respectively. This should be a way to improve the bandwidth.

Then, we observed the electric field distributions at different frequencies for the hybrid disk array in both the *x-y* and *x-z* plane. As shown in Figure 4d–f, when it works at a higher frequency 3.0 THz, the LSP mode is dominant; when it works at a lower frequency 1.55 THz, the coupling mode is dominant; when it works at the center frequency 2.3 THz, both modes exist, but neither is more dominant than the other. These results are consistent with the above analysis. In order to understand these three cases more clearly, the cross section plots of electric field distributions in the *x-z* plane are given in Figure 4g–i, with the corresponding *x-z* cross section shown in Figure 4j. It can be seen that as the frequency increases from 1.55 THz to 3.0 THz, the electric field gradually distributes from the edges to the whole disk, which further verify our analysis. Furthermore, because the the hybrid disk array is a combination of the larger and smaller disk arrays, along with the increase of graphene area, the disk spacing becomes smaller compared with the above two arrays, both the LSP mode and the coupling mode are enhanced, thus the absorption bandwidth is significantly expanded.

All the above observations are based on TE polarized wave incidence. Due to the symmetry of the absorber structure, the electric field distributions are the same for TE and TM polarized THz waves at the same frequency, there is only a 90 degree rotation symmetry difference between them.

The absorption phenomenon can be interpreted through the effective medium theory. As the underlying metal acts as a perfect reflective layer, resulting in $T=|S_{21}{|}^{2}\approx 0$, the relationship between effective impedance and absorptance is as follows [58]:

(6) $$A=1-\;R\left(\omega \right)=1-{\left|\frac{Z-Z_{0}}{Z+Z_{0}}\right|}^{2}=1-{\left|\frac{Z_{r}-1}{Z_{r}+1}\right|}^{2},$$

where *Z* is the effective impedance, $Z_{0}$ is the free space impedance and $Z_{r}=Z/Z_{0}$ is the relative impedance between the proposed absorber and the free space. It can be retrieved from the complex frequency dependent reflection and transmission coefficients using the following formula [58,59]:

(7) $$Z_{r}=\sqrt{\frac{{\left(1+S_{11}\right)}^{2}-{S_{21}}^{2}}{{\left(1-S_{11}\right)}^{2}-{S_{21}}^{2}}}.$$

**Figure 5:**
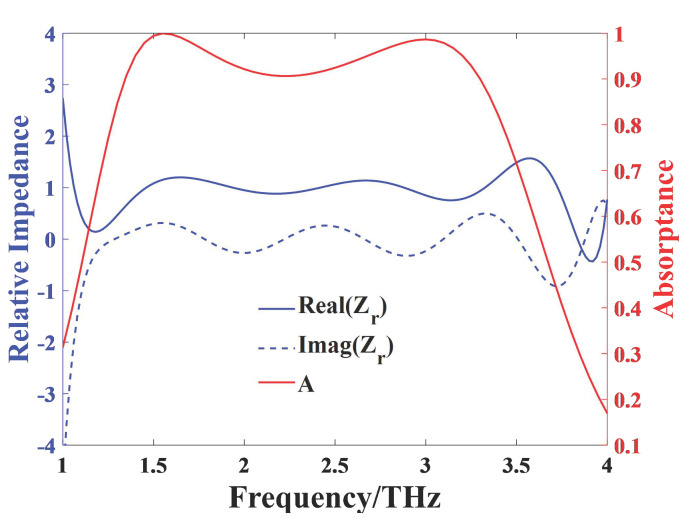
The relative impedance of the absorber in the case of normal incidence.

As shown in Figure 5, the blue solid curve and blue dot curve represent the real part Re($Z_{r}$) and the imaginary part Im($Z_{r}$) of the relative impedance $Z_{r}$, respectively. The results reveal that Re($Z_{r}$) is close to 1 and Im($Z_{r}$) is close to 0 between 1.3 THz and 3.4 THz, implying that *Z* nearly matches the impedance of free space. Therefore, a broadband absorption with high absorptance is achieved owing to the small reflectance and complete lack of transmission of the absorber in that frequency range.

**Figure 6:**
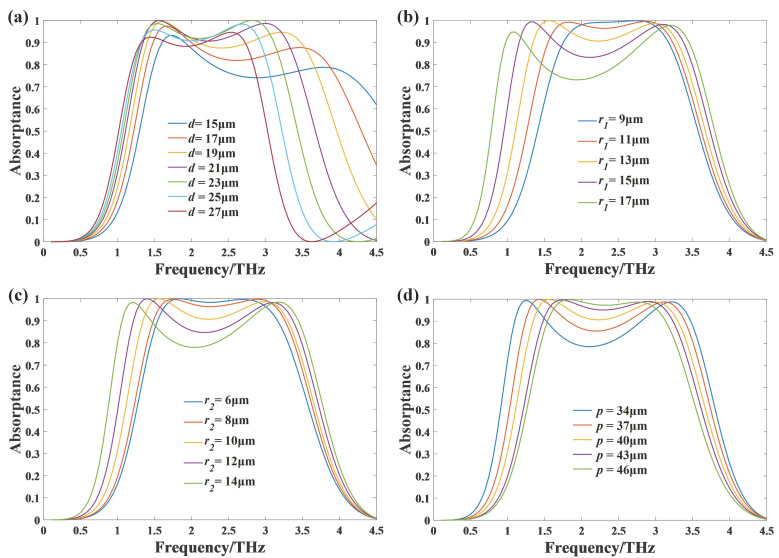
The simulation of absorptance of the absorber for geometric parameter sweep. (**a**) The results with different thickness *d* of the substrate; (**b**) the results with different radius $r_{1}$ of the larger disks; (**c**) the results with different radius $r_{2}$ of the smaller disks; (**d**) the results with different period *p*.

In order to further analyze the principle of broadband absorption, we make geometric parametric sweep analysis and discussion on the proposed absorber under the condition of normal incidence, and the results are shown in Figure 6. Figure 6a displays the effect of substrate thickness *d* on the absorption properties. It can be seen that with the increase of *d*, the absorption bandwidth decreases and the center frequency experiences a red shift. When *d* increases from 15μm to 21μm, the two absorption peaks rise significantly, so the absorption performance becomes better. When *d* continues to increase from 21μm to 27μm, the two absorption peaks both decrease. Considering bandwidth and absorption performance together, *d* has an optimal value 21 μm. This is because the impedance matching condition for eliminating reflection on the graphene surface is dependent on the thickness of the dielectric material, so the absorption is sensitive to the thickness of the dielectric. Figure 6b,c gives the effects of the two radii of the graphene circular patches on the frequency characteristics with all other parameters remaining the same as before. The results show that with the increase of $r_{1}$ or $r_{2}$, the lower frequency resonant point has an obvious red shift, while the higher frequency resonant point is blue-shifted slightly, and both absorption peaks maintain high absorptance. The reason is that when the radius gets larger, the spacing between each two adjacent patches gets smaller, so the coupling effect gets stronger, which is related to the lower frequency resonance. Meanwhile, larger radius means larger graphene area ratio, which is related to the higher frequency resonance. The superposition of these two different resonances leads to broadband absorption and a center frequency valley between the two peaks at both ends. Figure 6d shows the effect of the unit cell period *p*. As the period *p* decreases from 45 μm to 34 μm, the absorption bandwidth increases, but the absorptance near the center frequency decreases significantly. Decreasing the period *p* leads to smaller spacing and larger graphene area ratio, so the variation of the absorption characteristics is similar to that of increasing $r_{1}$ and $r_{2}$. With consideration of the performance of the absorption bandwidth and the absorptance intensity of the absorber, we finally optimized the size parameters as follows: d=21μm, $r_{1}=13\;\mu m$, $r_{2}=10\;\mu m$, p=40μm.

**Figure 7:**
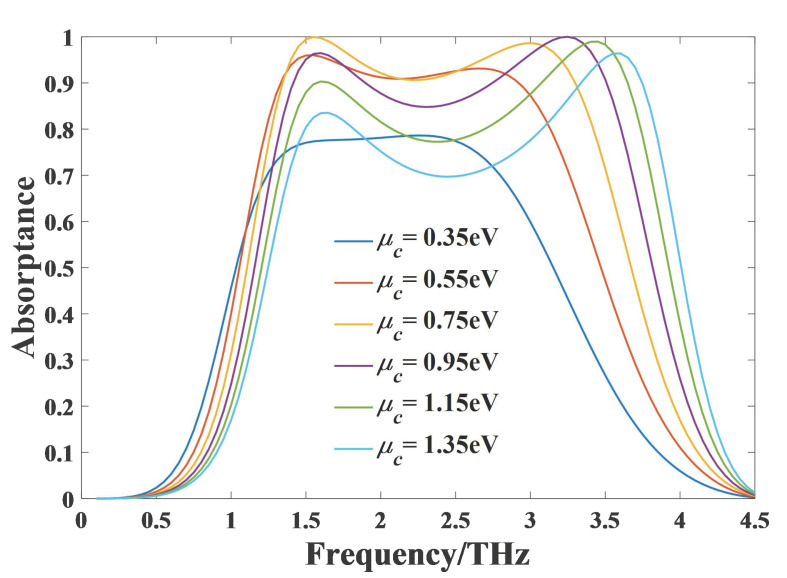
The simulated absorptance of the absorber for different chemical potentials.

In order to study the effect of the chemical potential of graphene, absorptance under different chemical potentials are plotted in Figure 7. When the chemical potential increases from 0.35 eV to 0.75 eV, the absorptance and absorption bandwidth increase gradually, so the absorption performance becomes better. When the chemical potential continues to increase from 0.75 eV to 1.35 eV, the absorption bandwidth increase and the center frequency has a blue shift, but the overall absorption significantly deteriorates, especially in the low frequency band. This phenomenon can also be explained by the impedance matching theory. We have compared the relative impedance under different chemical potentials. When $\mu_{c}$ = 0.75 eV, the real and imaginary parts of relative impedance are closest to 1 and 0 respectively in the broadband absorption range, which means that the impedance under this chemical potential matches that of free space best. From Figure 7, we can also notice that with the increase of $\mu_{c}$, the higher frequency resonance point has an obvious blue shift, which leads to the absorption bandwidth increase. The reason is that with the increase of $\mu_{c}$, equivalent inductance of the structure decreases, resulting in an increased resonance frequency [60]. So considering bandwidth and absorption performance together, $\mu_{c}$ has an optimal value 0.75 eV.

**Figure 8:**
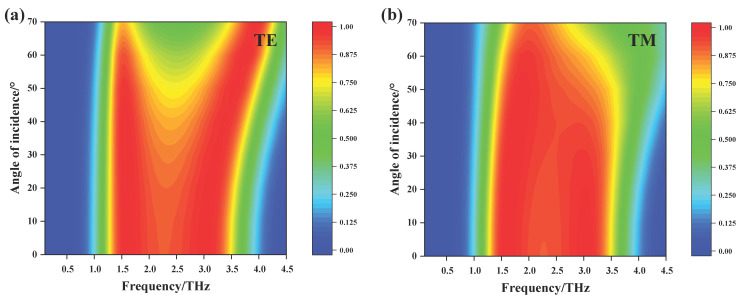
The simulated absorptance of the proposed absorber vary with frequency and oblique incident angle under TE (**a**) and TM (**b**) polarized waves incidence.

Finally, we analyze the influence of incident angle on the absorption characteristics of the proposed absorber. All the above simulations were carried out in the case of normal incidence, in which case the TE and TM polarized waves were equivalent because of the symmetry of the structure. However, in practical applications, the absorption characteristics of the absorber for oblique incident wave are more meaningful. Therefore, we analyzed the absorption performance of TE and TM polarized waves at different incident angles. Figure 8 shows the absorptance of the absorber under TE and TM polarized waves as a function of frequency and incident angle. Under TE polarized wave incidence, with the increase of incident angle, the absorptance at both the lower frequency resonance and the higher frequency resonance is still very high, but that near the center frequency is obviously weakened. Under TM polarized wave incidence, the absorptance remains stable when the incident angle is within 50°. When the incident wave is greater than 50°, the absorption bandwidth gradually decreases and the absorptance at the higher frequency resonance is weakened. In short, for both TE or TM polarized waves incidence, the absorber is polarization insensitive in the case of at a small incident angle. When the incident angle is in the range of 0° to 50°, the absorptance is greater than 70% within the operating frequency range, which ensures the device has a good performance with broad incident angle. This can be explained by the close correlation between near-unity absorption and graphene plasmon resonance, we can say the absorber is not very sensitive to the incident angle.

## 4. Conclusions

In summary, a polarization-insensitive broadband THz absorber based on single-layer graphene circular patches is proposed in this work. Numerical simulation results demonstrate that the optimized absorber has a bandwidth of 2 THz for over 90% absorptance, ranging from 1.34 THz to 3.36 THz. In addition, due to the rotational symmetry of the device structure, the absorber has perfect polarization independence, which has been verified by simulation. Moreover, it can achieve broadband absorption with absorptance up to 70% both at TE and TM polarized waves under wide angle incidence, ranging from 0° to 50°. Furthermore, note that by changing the chemical potential, the absorption bandwidth and absorptance of the absorber can be adjusted so as to achieve different absorption performance of the absorber. Not only does this absorber have excellent performance, but it also has the advantages of simple structure and easy processing. Benefiting from these advantages and promising performance, it can develop many potential applications in various fields, such as THz detection, imaging and cloaking objects and others.

## Data Availability

Data are available in the main text.

## References

[1] Tonouchi M. (2007). Cutting-edge THz technology. Nat. Photonics.

[2] Nikoo M.S., Jafari A., Perera N., Zhu M., Matioli E. (2020). Nanoplasma-enabled picosecond switches for ultrafast electronics. Nature.

[3] Delfanazari K., Klemm R.A., Joyce H.J., Ritchie D.A., Kadowaki K. (2020). Integrated, portable, tunable, and coherent terahertz sources and sensitive detectors based on layered superconductors. Proc. IEEE.

[4] Siegel P.H. (2002). Terahertz technology. IEEE Trans. Microw. Theory Tech..

[5] Risacher C., Güsten R., Stutzki J., Hübers H., Büchel D., Graf U.U., Heyminck S., Honingh C.E., Jacobs K., Klein B. (2016). First supra-THz heterodyne array receivers for astronomy with the SOFIA observatory. IEEE Trans. Terahertz Sci. Technol..

[6] Lee S.W. (1971). Scattering by dielectric-loaded screen. IEEE Trans. Antennas Propag..

[7] Woodward R. (2002). Terahertz pulse imaging in reflection geometry of human skin cancer and skin tissue. Phys. Med. Biol..

[8] Ajito K. (2015). Terahertz spectroscopy for pharmaceutical and biomedical Applications. IEEE Trans. Terahertz Sci. Technol..

[9] Federici J., Moeller L. (2010). Review of terahertz and subterahertz wireless communications. J. Appl. Phys..

[10] Zeng H., Zhang Y., Lan F., Liang S., Wang L. (2019). Terahertz dual-polarization beam splitter via an anisotropic matrix metasurface. IEEE Trans. Terahertz Sci. Technol..

[11] Zhao Y., Wang L., Zhang Y., Qiao S., Yang Z. (2019). High-speed efficient terahertz modulation based on tunable collective-individual state conversion within an active 3 nm two-dimensional electron Gas metasurface. Nano Lett..

[12] Cui T.J., Li L., Liu S., Ma Q., Cheng Q. (2020). Information metamaterial systems. iScience.

[13] He J., Xie Z., Sun W., Wang X., Ji Y., Wang S., Yuan L., Yan Z. (2016). Terahertz tunable metasurface lens based on vanadium dioxide phase transition. Plasmonics.

[14] Landy N.I., Bingham C.M., Tyler T., Jokerst N., Smith D.R., Padilla W.J. (2008). Design, theory, and measurement of a polarization insensitive absorber for terahertz imaging. Phys. Rev. B Condens. Matter Mater. Phys..

[15] Wilbert D.S., Hokmabadi M.P., Martinez J. (2014). Terahertz metamaterials perfect absorbers for sensing and imaging. Proc. SPIE.

[16] Carranza I.E., Grant J., Gough J., Cumming D. (2016). Uncooled CMOS terahertz imager using a metamaterial absorber and pn diode. Opt. Lett..

[17] Bosquespadilla F.J., Landy L.N., Smith W.K., Smith J.D., Padilla E., Sajuyigbe S., Mock D.J. (2008). Perfect metamaterial absorber. Phys. Rev. Lett..

[18] Chen H.T. (2012). Interference theory of metamaterial perfect absorbers. Opt. Express.

[19] Wen Q.Y., Zhang H.W., Xie Y.S., Yang Q.H., Liu Y.L. (2009). Dual band terahertz metamaterial absorber: Design, fabrication, and characterization. Appl. Phys. Lett..

[20] Yong M., Qin C., Grant J., Saha S.C., Cumming D. (2011). A terahertz polarization insensitive dual band metamaterial absorber. Opt. Lett..

[21] Cheng H., Chen S., Yang H., Li J., An X., Gu C., Tian J. (2012). A polarization insensitive and wide-angle dual-band nearly perfect absorber in the infrared regime. J. Opt..

[22] Shen X., Cui T.J., Zhao J., Ma H.F., Hui L. (2011). Polarization-independent wide-angle triple-band metamaterial absorber. Opt. Express.

[23] Pan W., Yu X., Zhang J., Zeng W. (2017). A broadband terahertz metamaterial absorber based on two circular split rings. IEEE J. Quantum Electron..

[24] Wen Y., Ma W., Bailey J., Matmon G., Yu X. (2015). Broadband terahertz metamaterial absorber based on asymmetric resonators with perfect absorption. IEEE Trans. Terahertz Sci. Technol..

[25] Wang B.X., Wang L.L., Wang G.Z., Huang W.Q., Zhai X. (2014). Theoretical investigation of broadband and wide-angle terahertz metamaterial absorber. IEEE Photonics Technol. Lett..

[26] Chen H.T., Padilla W.J., Zide J.M.O., Gossard A.C., Taylor A.J., Averitt R.D. (2006). Active terahertz metamaterial devices. Nature.

[27] Okada T., Tanaka K. (2011). Photo-designed terahertz devices. Sci. Rep..

[28] Zhang Y., Qiao S., Sun L., Shi Q.W., Huang W., Li L., Yang Z. (2014). Photoinduced active terahertz metamaterials with nanostructured vanadium dioxide film deposited by sol-gel method. Opt. Express.

[29] Jeong Y.G., Bernien H., Kyoung J.S., Park H.R., Kim H.S. (2011). Electrical control of terahertz nano antennas on *VO*_2_ thin film. Opt. Express.

[30] Gosciniak J., Tan D. (2013). Theoretical investigation of graphene-based photonic modulators. Sci. Rep..

[31] Shi S.F., Zeng B., Han H.L., Hong X., Tsai H.Z. (2015). Optimizing broadband terahertz modulation with hybrid graphene/metasurface structures. Nano Lett..

[32] Geim A.K., Novoselov K.S. (2007). The rise of graphene. Nat. Mater..

[33] Koppens F.H.L., Chang D.E., de Abajo F.J.G. (2011). Graphene plasmonics: A platform for strong light-matter Interactions. Nano. Lett..

[34] He X.Y., Tao J., Meng B. (2013). Analysis of graphene TE surface plasmons in the terahertz regime. Nanotechnology.

[35] Li Z.Q., Henriksen E.A., Jiang Z., Hao Z., Martin M.C., Kim P., Stormer H.L., Basov D.N. (2008). Dirac charge dynamics in graphene by infrared spectroscopy. Nat. Phys..

[36] Hanson G.W. (2008). Dyadic Green’s functions for an anisotropic, non-local model of biased graphene. IEEE Trans. Antennas Propag..

[37] Batrakov K., Kuzhir1 P., Maksimenko1 S. (2016). Enhanced microwave-to-terahertz absorption in graphene. Appl. Phys. Lett..

[38] Lobet M. (2015). Robust electromagnetic absorption by graphene/polymer heterostructures. Nanotechnology.

[39] Alaee R., Farhat M., Rockstuhl C., Lederer F. (2012). A perfect absorber made of a graphene micro-ribbon metamaterial. Opt. Express.

[40] Nikitin A.Y., Guinea F., Garcia-Vidal F.J., Martin-Moreno L. (2012). Surface plasmon enhanced absorption and suppressed transmission in periodic arrays of graphene ribbons. Phys. Rev. B.

[41] Ke S., Wang B., Huang H., Long H., Wang K., Lu P. (2015). Plasmonic absorption enhancement in periodic cross-shaped graphene arrays. Opt. Express.

[42] Dong Y., Liu P., Yu D., Li G., Yang L. (2016). A tunable ultra-broadband ultrathin terahertz absorber using graphene stacks. IEEE Antennas Wirel. Propag. Lett..

[43] Andryieuski A., Lavrinenko A.V. (2013). Graphene metamaterials based tunable terahertz absorber: Effective surface conductivity approach. Opt. Express.

[44] Zhang Y., Feng Y., Zhu B., Zhao J., Jiang T. (2014). Graphene based tunable metamaterial absorber and polarization modulation in terahertz frequency. Opt. Express.

[45] Chen M., Sun W., Cai J., Chang L., Xiao X. (2017). Frequency-tunable terahertz absorbers based on graphene metasurface. Opt. Commun..

[46] Su Z., Wang Y., Xin L., Hao L., Chao Z., Li M., Tian S., Yang G. (2018). A tunable THz absorber consisting of an elliptical graphene disk array. Phys. Chem. Chem. Phys..

[47] Wang F., Huang S., Li L., Chen W., Xie Z. (2018). Dual-band tunable perfect metamaterial absorber based on graphene. Appl. Opt..

[48] Li Y., Wu J., Wang C., Shen Z., Wu D. (2019). Tunable broadband metamaterial absorber with single-layered graphene arrays of rings and discs in terahertz range. Phys. Scr..

[49] Ye L., Chen X., Cai G., Zhu J., Na L., Liu Q. (2018). Electrically tunable broadband trahertz absorption with hybrid-patterned graphene metasurfaces. Nanomaterials.

[50] Ju L., Geng B., Horng J., Girit C. (2011). Graphene plasmonics for tunable terahertz metamaterials. Nat. Nanotechnol..

[51] Yan H., Li X., Chandra B. (2012). Tunable infrared plasmonic devices using graphene/insulator stacks. Nat. Nanotechnol..

[52] Mishra R., Sahu A., Panwar R. (2019). Cascaded graphene frequency selective surface integrated tunable broadband terahertz metamaterial absorber. IEEE Photonics J..

[53] Gusynin V.P., Sharapov S.G., Carbotte J.P. (2007). Magneto-optical conductivity in graphene. J. Phys. Condens. Matter.

[54] Hanson G.W. (2008). Dyadic Green’s functions and guided surface waves for a surface conductivity model of graphene. J. Appl. Phys..

[55] Vakil A., Engheta N. (2011). Transformation optics using graphene. Science.

[56] Xu W., Zhu Z.H., Liu K., Zhang J.F., Yuan X.D., Lu Q.S., Qin S.Q. (2015). Toward integrated electrically controllable directional coupling based on dielectric loaded graphene plasmonic waveguide. Opt. Lett..

[57] Ye L., Sui K., Liu Y., Zhang M., Huo L.Q. (2018). Graphene-based hybrid plasmonic waveguide for highly efficient broadband mid-infrared propagation and modulation. Opt. Express.

[58] Liu Y., Huang R., Ouyang Z. (2021). Terahertz absorber with dynamically switchable dual-broadband based on hybrid metamaterial with vanadium dioxide and graphene. Opt. Express.

[59] Ding F., Dai J., Chen Y., Zhu J., Jin Y., Bozhevolnyi S.I. (2016). Broadband near-infrared metamaterial absorbers utilizing highly lossy metals. Sci. Rep..

[60] Xiao B., Gu M., Xiao S. (2017). Broadband, wide-angle and tunable terahertz absorber based on cross-shaped graphene arrays. Appl. Opt..

